# Self-Blinking Dye Restores Efficient Use of Nanobodies in Single-Molecule Localization Microscopy

**DOI:** 10.1021/acs.nanolett.6c02799

**Published:** 2026-08-26

**Authors:** Samrat Basak, Kaushik Inamdar, Yoav G. Pollack, László Albert, Daniel C. Jans, Stefan Jakobs, Jörg Enderlein, Roman Tsukanov, Felipe Opazo

**Affiliations:** † III. Institute of Physics−Biophysics, Georg-August University, 37077 Göttingen, Germany; ‡ Department of Chemistry and Center for NanoScience, Ludwig-Maximilians-Universität München, Butenandtstraße 5−13, 81377 München, Germany; § Department of NanoBiophotonics, 28282 Max Planck Institute for Multidisciplinary Sciences, 37077 Göttingen, Germany; ∥ Department of Neurology, University Medical Center Göttingen, 37073 Göttingen, Germany; ⊥ Institute for the Dynamics of Complex Systems, Georg-August University, 37077 Göttingen, Germany; # Center for Biostructural Imaging of Neurodegeneration, 27177 University Medical Center Göttingen, 37075 Göttingen, Germany; ¶ Institute of Neuro- and Sensory Physiology, 27177 University Medical Center Göttingen, 37073 Göttingen, Germany; $ Translational Neuroinflammation and Automated Microscopy, Fraunhofer Institute for Translational Medicine and Pharmacology (ITMP), 37073 Göttingen, Germany; ○ Cluster of Excellence “Multiscale Bioimaging: from Molecular Machines to Networks of Excitable Cells”, Georg-August University, 37075 Göttingen, Germany; ∇ Multiscale Biology, Faculty of Biology and Psychology, Georg-August-University Göttingen, 37077 Göttingen, Germany; ◆ NanoTag Biotechnologies GmbH, 37079 Göttingen, Germany

**Keywords:** Self-blinking fluorophores, Nanobodies, Fluorophore photophysics, Single-molecule localization microscopy, dSTORM, MINFLUX

## Abstract

Direct stochastic optical reconstruction microscopy (dSTORM) relies on controlled fluorophore blinking to achieve nanometer-scale resolution, yet the field’s benchmark dye, Alexa Fluor 647, underperforms when conjugated to nanobodies, limiting the practical use of minimal-linkage labeling strategies. Here, we show that the self-blinking dye JF635b overcomes this limitation by maintaining robust, photostable blinking upon conjugation to nanobodies under buffer-independent conditions. This enables reliable single-molecule localization microscopy (SMLM) without the need for complex switching buffers. Using JF635b-labeled nanobodies, we demonstrate consistent performance across multiple imaging modalities, including wide-field dSTORM, fluorescence-lifetime SMLM, and MINFLUX nanoscopy, achieving localization precisions from ∼15 nm down to the subnanometer regime. In addition, JF635b supports long-term sample preservation and efficient blinking even in pure water, enabling minimally perturbative imaging conditions. Together, these results establish self-blinking dSTORM as a robust and accessible platform for quantitative nanoscopy across experimental contexts.

## Graphical abstract

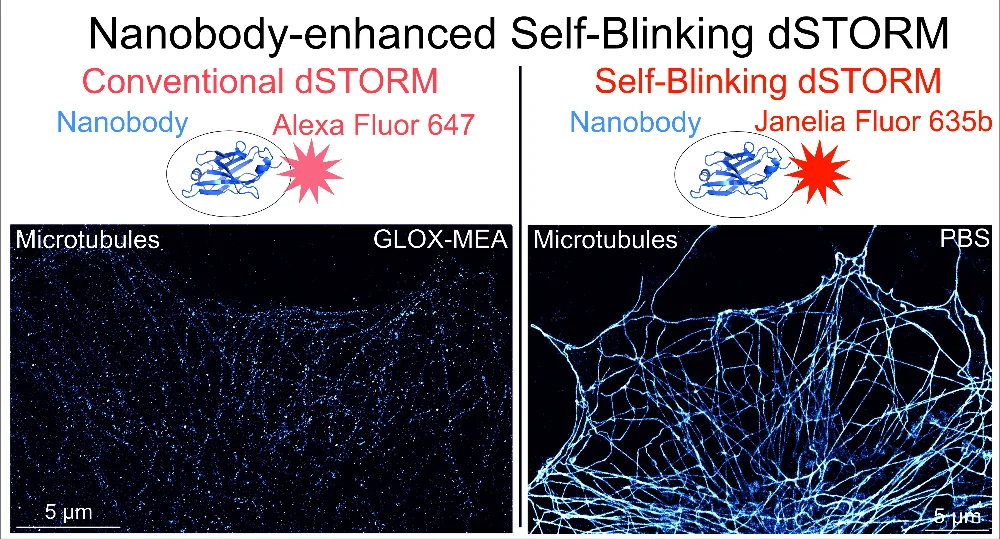

Single-molecule localization microscopy (SMLM)^1,2^ enables nanometer-scale localization precision. Among its core implementations, direct stochastic optical reconstruction microscopy (dSTORM)^3^ routinely achieves localization precision below 20 nm, but performance critically depends on the fluorophore blinking characteristics. Alexa Fluor 647 (AF647) has long been the benchmark dye, exhibiting robust blinking in buffers such as glucose oxidase with catalase (GLOX) with primary thiol (MEA).^3,4^ Careful optimization of blinking buffer composition for each sample is required, especially in multitarget imaging, which often requires compromises, leading to the development of sequential-labeling strategies such as NanoPlex^5^ or microfluidics-enhanced Exchange-PAINT.^6^

Despite its widespread adoption, dSTORM remains highly sensitive to imaging conditions, with fluorophore blinking typically requiring carefully optimized chemical environments. Small variations in buffer composition, oxygen-scavenging efficiency, or reducing agents can substantially affect blinking behavior, localization density, and photostability,^7,8^ posing a major challenge to reproducibility across laboratories and imaging facilities. These constraints are particularly problematic for multitarget imaging, long acquisition times, and workflows involving repeated imaging or sample storage.

Linkage error in indirect immunofluorescence (IF) labeling occurs when the primary antibody (1.Ab) targeting the protein of interest (POI) is labeled with a fluorescent secondary antibody (2.Ab). Indirect IF displaces fluorophores by up to 25 nm from the POI. Nanobodies (Nbs) reduce this linkage error to approximately 3 nm, making them ideal probes^9^ for SMLM nanoscopy. However, a practical limitation has emerged: when directly conjugated to nanobodies, commonly used dyes such as AF647 often exhibit compromised blinking behavior and reduced photostability. This undermines the very advantages nanobodies offer for high-precision SMLM and restricts their use in workflows that rely on consistent and sustained photoswitching.

Self-blinking dyes provide an elegant solution by spontaneously cycling between bright and dark states without the need for blinking-reducing buffers. Several such fluorophores have been explored for super-resolution microscopy, including specialized dyes for live-cell super-resolution imaging,^10^ Thioflavin T for fibrils,^11^ rhodamine–coumarin hybrids,^12^ silicon rhodamine analogues for STORM–MINFLUX,^13^ and rhodamine-based dyes for super-resolution optical fluctuation imaging.^14^

JF635b is a newly developed, spontaneously blinking rhodamine from the hydroxymethyl-SiR Janelia Fluor series, described in Holland et al.^15^ It exhibits an excellent duty cycle, operates without reducing blinking buffers, and is compatible with diverse SMLM labeling strategies. Although its photophysical properties have been characterized when conjugated to HaloTags,^15^ its potential for robust nanobody-based SMLM has not been systematically explored. Here, we benchmark JF635b against AF647 across Ab and Nb conjugates using wide-field self-blinking dSTORM (sb-dSTORM), confocal fluorescence-lifetime SMLM (FL-SMLM),^16–18^ and MINFLUX.^19,20^

To define how fluorophore choice impacts the performance of nanobody-based SMLM, we benchmarked the blinking behavior of AF647 and the self-blinking dye JF635b in a direct dSTORM implementation. COS-7 cells expressing TOMM70–EGFP–ALFA were immunostained using two widely applied nanobodies, NbGFP (binding EGFP)^9^ and NbALFA, part of the versatile ALFA-tag/NbALFA labeling system,^21^ each conjugated to either AF647 or JF635b. Samples labeled with AF647 were imaged under standard GLOX–MEA switching conditions, whereas JF635b-labeled samples were imaged in phosphate-buffered saline (PBS) without the use of chemical blinking buffers (Figure 1).

**Figure 1:**
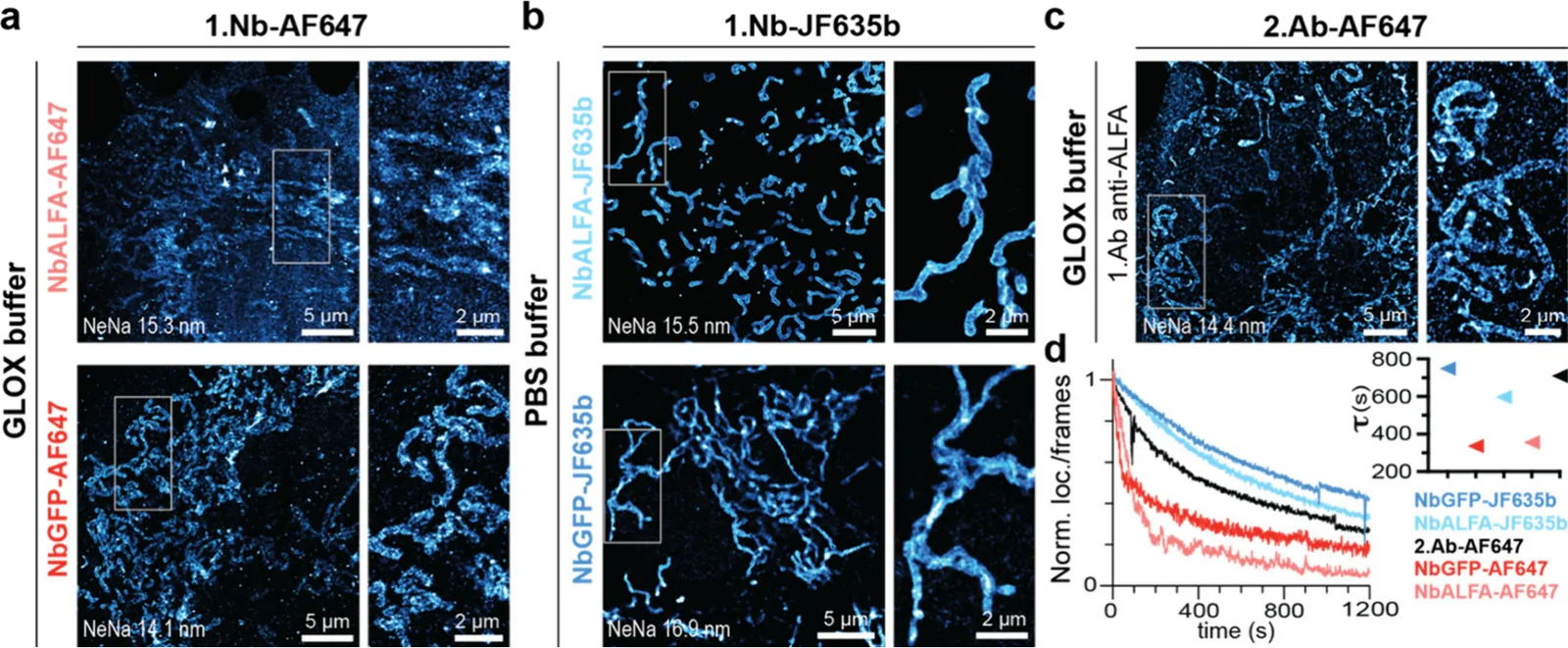
dSTORM using nanobodies conjugated with AF647 or JF635b. Primary nanobody (1.Nb) NbALFA and NbGFP, each conjugated with 2 AF647 (a) or JF635b (b), were used to directly label transfected COS-7 cells displaying EGFP and ALFA-tag on their mitochondria. (c) As a control, conventional 1.Ab anti-ALFA-tag, was revealed by secondary antibodies (2.Ab-AF647). Samples with AF647 were imaged under GLOX–MEA buffer, while samples with JF635b were imaged in PBS. Average localization precisions (NeNa) are indicated on the images. (d) Localization per frame was plotted, with the blinking differences between conditions shown as τ for each curve (details are provided in the Supporting Information).

Nanobodies conjugated to AF647 exhibited strongly impaired blinking behavior, characterized by reduced photon yields, shortened on-times, and rapid photobleaching, with most emitters bleaching within approximately 5 min of acquisition in wide-field SMLM under optimal imaging conditions (Figure S1 and Table S1). Compared to a control condition using conventional indirect IF to detect the ALFA-tag, NbALFA–AF647 conjugates produced ∼70% fewer localizations, an effect consistently observed for both NbGFP and NbALFA. Although both Nbs display comparable binding affinities, NbGFP–AF647 yielded marginally better reconstructions than NbALFA–AF647, while NbALFA–AF647 showed particularly poor blinking and rapid signal loss, resulting in clearly suboptimal mitochondrial reconstructions (Figure 1a,b). Importantly, these differences were independent of the target interaction and were consistently observed across nanobody types, indicating that the reduced performance originates from the dye–nanobody conjugate rather than from binding affinity or labeling efficiency.

In contrast, Nbs conjugated to JF635b exhibited stable, sustained self-blinking behavior when imaged in PBS. Individual emission bursts yielded 373 ± 169 photons with on-times of 35 ± 3 ms and remained detectable over tens of thousands of frames without appreciable photobleaching (Table S2 and Supplementary Movies 1, 2, 3, 4, and 5). Although individual blinking events were shorter-lived than those observed for AF647 under reducing conditions, the markedly higher number of blinking cycles resulted in higher localization densities, enabling faster and more complete reconstructions. As a result, JF635b-labeled Nbs produced continuous, densely sampled super-resolution images with localization precision comparable to or exceeding that obtained with conventional antibody-based labeling, confirming the high photostability and robustness of JF635b under buffer-independent sb-dSTORM conditions (Figure 1a–d and Table S1).

To test whether buffer-independent self-blinking nanobody probes are compatible with widely used antibody-based labeling workflows, we next examined secondary nanobody (2.Nb) detection strategies, which offer flexibility, multiplexing capacity, and broad applicability across established primary antibodies.^22^ Microtubules in COS-7 cells were labeled using a monoclonal anti-α-tubulin primary antibody, revealed with secondary nanobodies conjugated to two JF635b molecules per nanobody, and imaged by wide-field sb-dSTORM in PBS (Figures 2 and S2 and Supplementary Movie 6).

**Figure 2:**
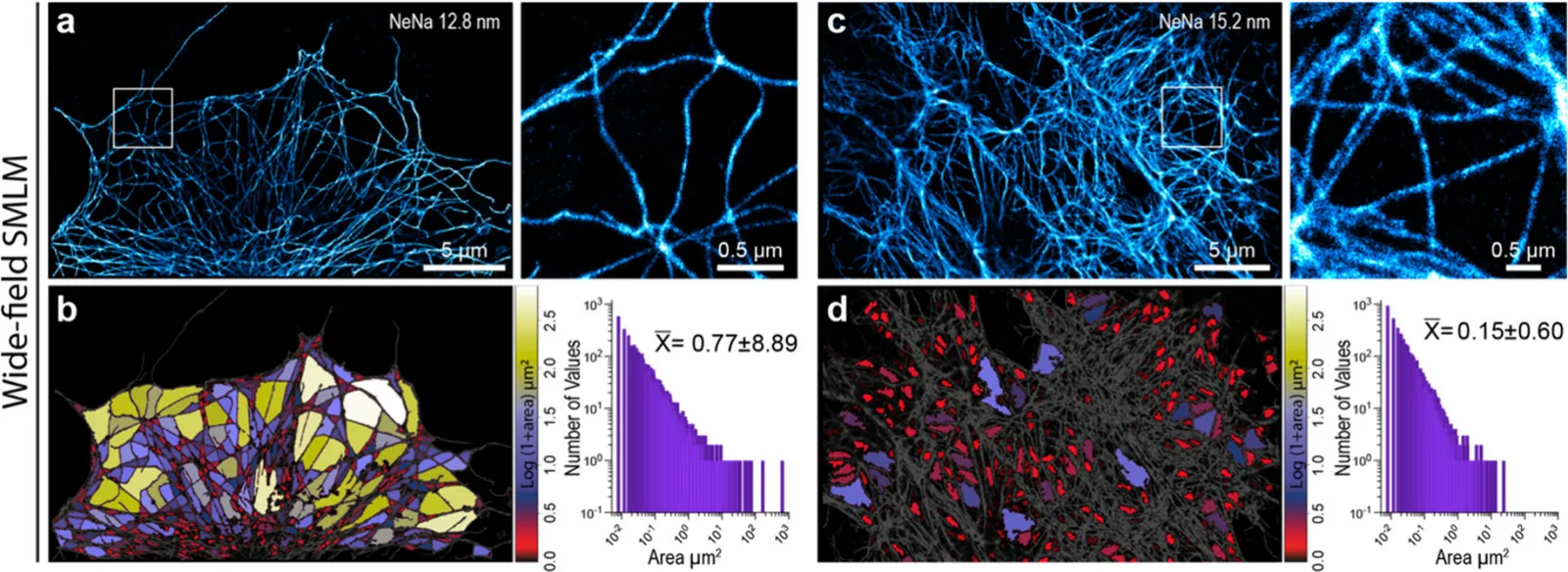
Applications of sb-dSTORM across labeling strategies and SMLM modalities. (a–d) Wide-field sb-dSTORM in COS-7 cells labeled with 1.Ab and 2.Nb-JF635b. (a and c) Full reconstruction with zoom-ins of microtubule and vimentin. (b and d) Segmentation analysis for parts a and c and area histograms for all data sets combined (microtubule and vimentin, respectively). Average area (*x̅*).

JF635b-labeled 2.Nbs yielded continuous and densely sampled reconstructions of microtubule filaments with a mean localization precision of 12.8 ± 4.5 nm (Figure 2a). The high localization density and filament continuity enabled reliable segmentation and quantitative analysis of the filament network (Figure 2b; segmentation workflow in Figure S3) demonstrating the suitability of sb-dSTORM data for uncomplicated quantitative image analysis. Applying the same labeling strategy to vimentin intermediate filaments using a polyclonal rabbit primary antibody produced similarly robust reconstructions with a localization precision of 15.2 ± 4.7 nm (Figure 2c). Quantitative segmentation analysis could be readily implemented for both filamentous structures despite their distinct network organizations (Figures 2d and S4 and Supplementary Movie 7). Comparable high labeling efficiency and image quality were observed for additional targets, including peroxisomes (Figure S5), further underscoring the versatility and broad applicability of this approach.

To assess long-term stability, a single independently prepared COS-7 sample labeled with JF635b-conjugated nanobodies was stored in PBS at 4 °C and reimaged after extended periods. At each time point, 6–10 individual cells were imaged to evaluate the temporal stability and robustness of labeling rather than sample-to-sample reproducibility. Over storage times of up to one year, JF635b-labeled nanobodies maintained stable blinking behavior and localization precision, with only a modest reduction in labeling density observed after 12 months (Figure 3a). In contrast, AF647 typically exhibits a significant decay in blinking performance within weeks. The pronounced stability of Nb-JF635b conjugates enables long-term sample preservation, repeated reimaging, and high-throughput data acquisition without the need for buffer exchange or relabeling.

**Figure 3:**
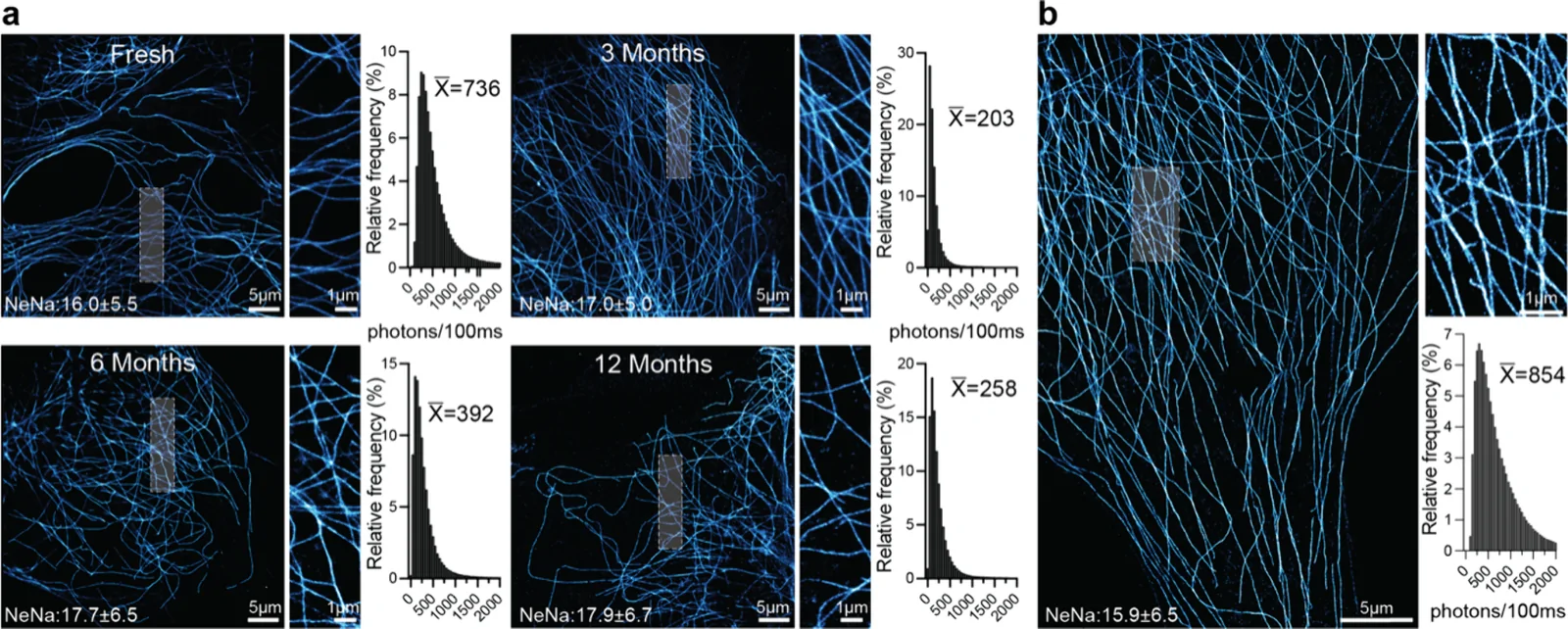
Long-term storage of the sample and the water performance of the Nb-JF635. (a) dSTORM images of the same sample imaged immediately after labeling (Fresh) and after 3, 6, and 12 months of storage at +4 °C in PBS. Blinking performance and localization precision (NeNa) as well as the photon output of 2.Nb-JF635b remained stable throughout the 12 months. Above the distributions of photons/100 ms, the average (*x̅*) is displayed. (b) dSTORM image of microtubules labeled with JF635b and imaged in pure water. The average localization precision (NeNa) and photon output were comparable to or higher than those in PBS.

We next asked whether the intrinsic blinking behavior of JF635b could support SMLM under conditions that are typically incompatible with conventional dSTORM fluorophores. Remarkably, 2.Nb-JF635b conjugates exhibited stable, reversible blinking even when imaged in pure water, in the complete absence of salts or additives (Figure 3b and Supplementary Movie 8). Despite these minimally perturbed conditions, localization densities and reconstruction quality remained sufficient for reliable super-resolution imaging, highlighting the exceptional robustness of the self-blinking mechanism and its compatibility with chemically sensitive workflows such as expansion microscopy (ExM).^23^

Because different SMLM modalities impose distinct constraints on fluorophore photophysics, photon budgets, and temporal dynamics, we next evaluated whether self-blinking nanobody probes could serve as a general labeling strategy across complementary localization techniques.

Using the same antibody–secondary nanobody labeling strategy as for wide-field sb-dSTORM, we performed MINFLUX^19^ imaging of microtubules and vimentin filaments labeled with JF635b-conjugated nanobodies. Under identical sample preparation conditions,^24^ JF635b-supported MINFLUX localization yielded mean combined localization precisions of 0.90 nm for microtubules and 0.97 nm for vimentin (Figure 4a,b), demonstrating that the spontaneous blinking kinetics of JF635b are fully compatible with high-precision MINFLUX localization.

**Figure 4:**
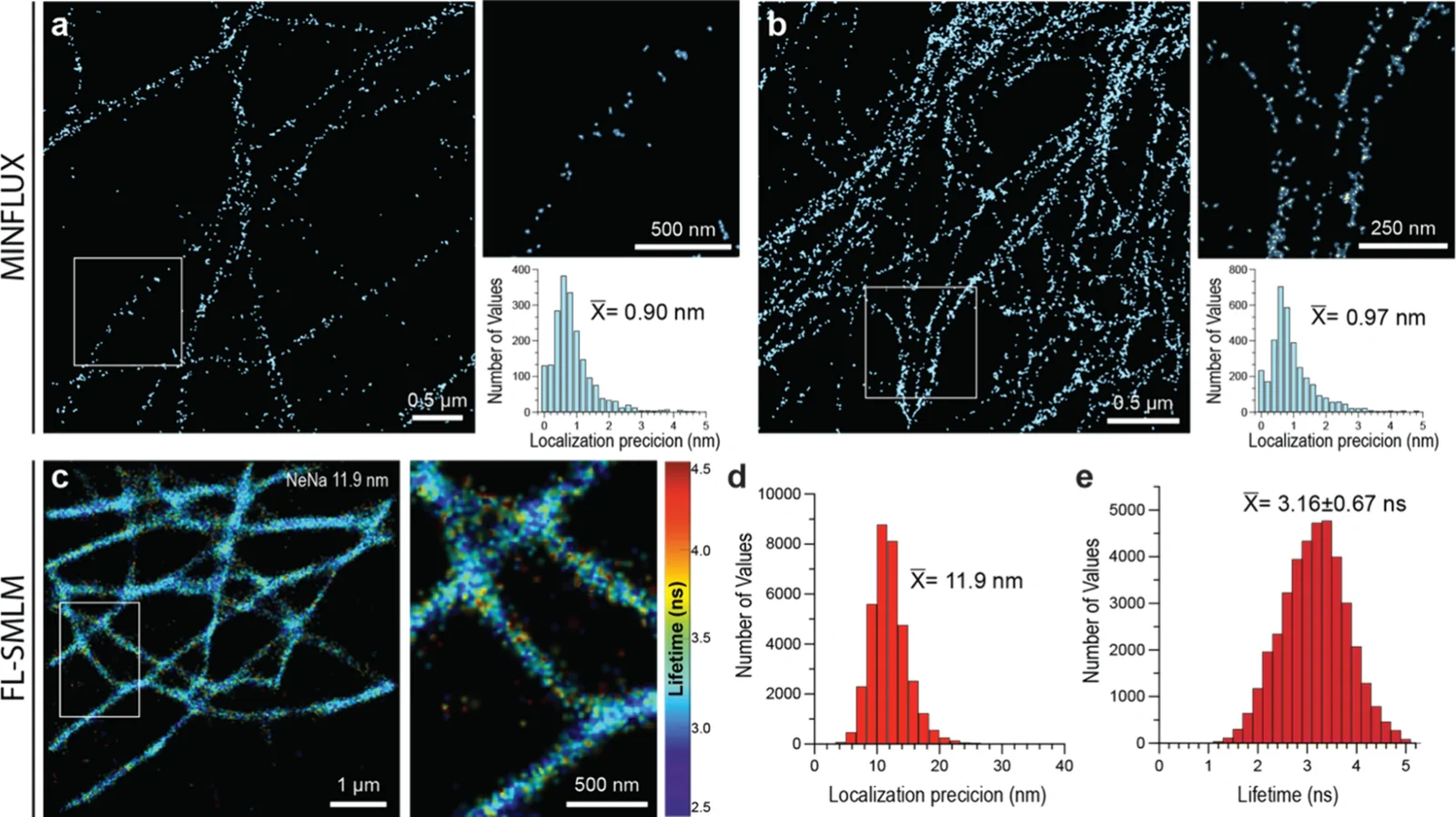
Evaluating JF635b in MINFLUX and FL-SMLM. MINFLUX imaging of the microtubule (a) and vimentin (b) staining using 2.Nb-JF635b. Combined localization precision plots yield mean precisions of 0.90 nm (microtubules) and 0.97 nm (vimentin). (c) Confocal FL-SMLM imaging of microtubules labeled with 1.Ab and 2.Nb-JF635b, (d) localization precision histogram, and (e) fluorescence-lifetime distribution (mean ± s.d. indicated).

We further tested the applicability of self-blinking nanobody probes in FL-SMLM^16^ using confocal laser scanning microscopy. JF635b-labeled microtubules produced high-quality lifetime-resolved super-resolution reconstructions with a mean localization precision of 11.9 nm and a fluorescence lifetime of 3.16 ± 0.67 ns (Figure 4c–e). These results demonstrate that JF635b is fully compatible with confocal SMLM while simultaneously providing robust lifetime information, enabling applications such as lifetime-based multiplexing.^17,25^

Together, these data demonstrate that self-blinking nanobody probes are compatible with a broad range of SMLM implementations, spanning wide-field, confocal, and MINFLUX-based localization microscopy and can serve as a unified labeling strategy across modalities.

dSTORM relies critically on fluorophore blinking, yet most implementations remain dependent on specialized chemical environments that limit robustness, reproducibility, and long-term sample stability. Although nanobodies offer clear advantages in labeling accuracy and epitope accessibility, reducing linkage error from ∼20–25 nm for conventional antibodies to ∼3 nm, their practical use in SMLM has been constrained by the poor blinking performance of commonly used dyes when directly conjugated. Our results identify fluorophore blinking as a central bottleneck in nanobody-based dSTORM and demonstrate that this limitation can be effectively overcome using self-blinking fluorophores.

By conjugating nanobodies to the spontaneously blinking dye JF635b, we enable robust and sustained localization microscopy under buffer-independent conditions. Self-blinking nanobody probes restore high localization densities and nanometer-scale precision for both direct and secondary labeling strategies, eliminating the need for reducing or oxygen-scavenging buffers. This simplification not only enhances experimental robustness but also lowers technical barriers, particularly in multiuser imaging facilities and high-throughput settings where reproducibility across experiments and users is critical.

A defining feature of JF635b-labeled nanobodies is their exceptional photophysical stability. Stable blinking in pure water and sustained performance after long-term storage for up to one year highlight the intrinsic robustness of the self-blinking mechanism. In contrast to conventional dSTORM approaches, where reactive imaging buffers degrade over time and introduce variability, samples labeled with JF635b can be stored in simple PBS and reimaged over extended periods with minimal loss of performance. This enables repeated imaging of the same specimen under comparable conditions, supporting longitudinal studies, large-scale comparisons, and the development of standardized reference samples. By removing buffer-related variability, self-blinking dSTORM directly addresses a major limitation in SMLM: reproducibility across samples and laboratories.

Beyond long-term stability, JF635b can sustain efficient blinking even in pure water, expanding SMLM’s operational range. Unlike conventional fluorophores that require carefully tuned environments, JF635b blinks reversibly in the absence of salts or additives, enabling imaging with minimal perturbation and simplifying workflows. This also opens opportunities for integration with chemically sensitive techniques such as expansion microscopy, where buffer composition affects structural preservation.^26^ Self-blinking nanobody probes work across various SMLM methods, including wide-field self-blinking dSTORM, FL-SMLM, and MINFLUX, including DNA-PAINT MINFLUX implementations,^20^ achieving subnanometer localization precision not only on commercial instruments but also on the custom-built pMINFLUX platform.^27^ Their cross-modality compatibility makes them a unifying labeling strategy for super-resolution microscopy, providing consistent probe behavior across platforms.

Overall, self-blinking nanobody probes combine minimal linkage error, photophysical robustness, and operational simplicity. By restoring the practical advantages of nanobody labeling and removing buffer dependence, they provide a broadly enabling solution that enhances reproducibility, flexibility, and accessibility in nanoscale super-resolution microscopy.

In summary, self-blinking nanobody probes combine minimal linkage error, photophysical robustness, and operational simplicity. By restoring the practical advantages of nanobody labeling and removing buffer dependence, they provide a broadly enabling solution that enhances reproducibility, flexibility, and accessibility in nanoscale super-resolution microscopy. While the molecular mechanism underlying the markedly different blinking behaviors of AF647 upon nanobody conjugation remains to be elucidated, our results establish JF635b as a versatile and robust probe for quantitative nanoscopy across multiple imaging modalities. Understanding the interplay between fluorophore photophysics, protein microenvironment, and blinking behavior represents an exciting direction for future studies and may provide valuable design principles for the next generation of self-blinking probes.

## Supplementary Material



















## Data Availability

All data supporting the conclusions of this study are available from the corresponding authors upon reasonable request. FiJi: https://fiji.sc/. Fiji ThunderSTORM plugin: https://github.com/zitmen/thunderstorm. TrackNTrace: https://github.com/NRadmacher/TrackNTrace/tree/ISM. Abberior Imspector is commercially available from Abberior Instruments GmbH, Göttingen, Germany.

## Notes

Experimental sections, wide-field SMLM, microtubules labeled with antimouse IgG1-JF635b–Images library, quantitative segmentation analysis of filamentous networks, vimentin filaments labeled with antirabbit nanobody-JF635b–Images library, peroxisomes (PMP70) labeled with antirabbit nanobody-JF635b–Images library, quantitative characterization of the fluorophore performance, antibodies and nanobodies used in this study, confocal Fl-SMLM, and MINLFLUX imaging parameters (PDF)
Supplementary Movie 1 (PDF)
Supplementary Movie 2 (MP4)
Supplementary Movie 3 (MP4)
Supplementary Movie 4 (MP4)
Supplementary Movie 5 (MP4)
Supplementary Movie 6 (MP4)
Supplementary Movie 7 (MP4)
Supplementary Movie 8 (MP4)
