# Supplementary material for: Self-Blinking Dye Restores Efficient Use of Nanobodies in Single-Molecule Localization Microscopy
Source: Nano Lett. 2026 Aug 26;26(35):11808–14. doi: 10.1021/acs.nanolett.6c02799 (PMC13564880; doi:10.1021/acs.nanolett.6c02799)
Supplement: Supplementary file 1 [file nl6c02799_si_002.pdf]

Supplementary Information

Self-blinking dye restores efficient use of nanobodies in single-molecule localization microscopy

Samrat Basak[1,2], Kaushik Inamdar[3,4], Yoav G. Pollack[5], László Albert[6,7], Daniel C. Jans[3,4], Stefan Jakobs[3,4,8,9], Jörg Enderlein[1,9], Roman Tsukanov[1,10,*] and Felipe Opazo[6,7,11,*]

1. III. Institute of Physics - Biophysics, Georg-August University, 37077 Göttingen, Germany.
2. Department of Chemistry and Center for NanoScience, Ludwig-Maximilians-Universität München, Butenandtstr. 5-13, 81377 München, Germany.
3. Department of NanoBiophotonics, Max Planck Institute for Multidisciplinary Sciences, 37077 Göttingen, Germany.
4. Department of Neurology, University Medical Center Göttingen, 37073 Göttingen, Germany.
5. Institute for the Dynamics of Complex Systems, Georg-August University, 37077 Göttingen, Germany.
6. Center for Biostructural Imaging of Neurodegeneration (BIN), University Medical Center Göttingen, 37075 Göttingen, Germany.
7. Institute of Neuro-and Sensory Physiology, University Medical Center Göttingen, 37073 Göttingen, Germany.
8. Translational Neuroinflammation and Automated Microscopy, Fraunhofer Institute for Translational Medicine and Pharmacology ITMP, 37073 Göttingen, Germany.
9. Cluster of Excellence “Multiscale Bioimaging: from Molecular Machines to Networks of Excitable Cells” (MBExC), Georg-August University, 37075 Göttingen, Germany.
10. Multiscale Biology, Faculty of Biology and Psychology, Georg-August-University Göttingen, 37077 Göttingen, Germany.
11. NanoTag Biotechnologies GmbH, 37079 Göttingen, Germany.

* Corresponding Authors: fopazo@gwdg.de; rtsukan@gwdg.de

Experimental sections

Cell culture

COS-7 cells were cultured in Petri dishes at 37 °C with 5% CO2 inside a humidified incubator. They were grown in complete Dulbecco's Modified Eagle Medium (DMEM, ThermoFisher Scientific), supplemented with 10% fetal bovine serum (FBS, ThermoFisher), 4 mM L-glutamine, and 1% penicillin/streptomycin (ThermoFisher). For sample preparation, cells were seeded onto 18 mm poly-L-lysine-coated coverslips in 12-well plates and incubated at 37 °C with 5% CO2 in a humidified environment. For transfection, a plasmid encoding TOM70-EGFP-ALFA-tag driven by a CMV promoter was mixed with Lipofectamine 2000 (Thermo Fisher) and added to the seeded cells according to the manufacturer's instructions. Transfection and expression were typically left for 16-18h, before cell fixation.

Immunostaining of cells

COS-7 cells were first treated for 30 s with pre-warmed Extraction Buffer (EB, 10 mM MES, 138 mM KCl,3 mM $MgCl_2$, 2 mM EGTA, 320 mM sucrose, pH 6.8) supplemented with 0.1% Saponin. This was directly followed by fixation in a pre-warmed solution of 4% paraformaldehyde and 0.1% Glutaraldehyde in EB at RT for 15 min. After removing the fixatives and rinsing with PBS, unreacted aldehyde groups were quenched using 100 mM glycine in PBS for 15 min. Cells were blocked and permeabilized with 3% serum albumin (BSA) and 0.1% Triton X-100 (Sigma-Aldrich) in PBS for 30 min at RT. For Fig. 1, NbGFP and NbALFA labeled with AF647 or JF635b were purchased as FluoTag®-X2 from NanoTag Biotechnologies (clone 1H1, Cat#: N0304; Cat#: N1502) and used at a 1:1000 dilution for 45 minutes. Recombinant anti-ALFA antibody carrying a mouse IgG1 Fc domain (NanoTag Biotechnologies, Cat# N1582) was used at a 1:500 dilution and detected by Goat anti-Mouse IgG (H+L) Cross-Adsorbed Secondary Antibody, Alexa Fluor™ 647, also used in a 1:500 dilution (ThermoFisher, Cat# A-21235). Microtubules and vimentin structures were labeled using a one-step immunofluorescence[1] with a mouse monoclonal (SySy, Cat# 302211) and a rabbit monoclonal (Abcam, Cat# ab92547), respectively. Secondary detection was performed using FluoTag®-X2 anti-Mouse IgG1 (Cat# N2002) or FluoTag®-X2 anti-Rabbit IgG (Cat# N2402). See Supp. Table S3 for details on the used Abs and Nbs. All samples, after staining and washing, were post-fixed in 4% PFA for 15-20 minutes at room temperature, the fixative was removed, and the samples were quenched again with 100 mM glycine in PBS.

Wide-field sb-dSTORM imaging and data analysis

Wide-field sb-dSTORM measurements were performed on a custom-built setup as described previously[2] and in the Supplementary Information (Supp Figure S1). Regions of interest (ROIs) of up to 50 × 50 µm² were recorded with a pixel size in the object plane of 103.5 nm, an exposure time of 30 ms, and an EMCCD gain of 500. Each dataset consisted of 50,000 frames. The laboratory temperature was maintained at 23.0°C to ensure thermal stability of the setup. Linear sample drifts of typically 1-2 pixels were observed during acquisition and were corrected by a cross-correlation algorithm. Excitation was performed using a highly inclined and laminated optical sheet (HILO) configuration with a laser power of ~20 mW, yielding an optimal signal-to-noise ratio. Data analysis has been performed using free software FiJi[3], plugin ThunderSTORM[4]. Same analysis parameters were applied as in previous works[2,5]. Photostability was quantified by tracking the number of localizations per frame over time (Fig. 1d). Localization tables were exported from the ThunderSTORM plugin as .csv files and processed with a custom Python script to generate photostability plots (cumulative localizations in time, see Fig. 1d). The photostability curves were fitted with an exponential decay, from which characteristic photobleaching times were extracted. Segmentation of reconstructed super-resolution images was carried out using a standard Python image pipeline. Images were preprocessed using intensity filtering and morphological operations to ensure filament continuity and remove non-filament objects, and then inverted to enable analysis of voids rather than filaments. Void regions were then identified (and scaled to 10.35 nm/pixel), border-adjacent partial objects were removed, and regions were hole-filled to avoid biasing the measurement of their spacing area. Quantitative parameters, including void area distributions, were extracted (see Supplementary Information (Fig. S3) for details).

Confocal FL-SMLM sb-dSTORM imaging and data analysis

CLSM measurements were performed using a custom-built confocal microscope as described elsewhere[6,7] and in the Supplementary Information (Fig. S6). Regions of interest (ROIs) of 7 × 7 µm² were scanned, each subdivided into 70 × 70 virtual pixels (100nm/pixel). Similar image acquisition parameters were used as described previously[6]. During analysis, temporal binning of four consecutive frames was applied to improve signal-to-noise ratio. Data analysis has been performed using custom Matlab-based software TrackNTrace LifetimeEdition[6], freely available at https://github.com/scstein/TrackNTrace/releases/tag/v2.0.

MINFLUX sb-dSTORM imaging

MINFLUX sb-dSTORM measurements were performed on a commercial MINFLUX nanoscope (Abberior Instruments GmbH, Göttingen) controlled by *Imspector* software. Data were acquired using the standard 2D acquisition protocol described by Sahl *et al.*[8], *see Supplementary Table S4 for details*. Gold colloid particles (150 nm; BBI Solutions, catalogue no. EM.GC150/4, Crumlin, UK) were added to the samples as fiducial markers for active beam-path drift correction.

Regions of interest (ROIs) of 5 × 5 µm² were acquired over a total measurement time of ~6 h. Excitation was performed with the 642 nm laser at 5-10% output (~25-50 µW) as part of the MINFLUX localization routine. Image data were saved as MSR files and processed in MATLAB (MathWorks, USA).

Localization precisions were calculated as described previously[9]. In brief, localizations from binding events with more than four detections were combined. For enhanced visualization, MINFLUX images were rendered in Blender 3.4.1: raw localizations were displayed as overlapping blue spheres (20 nm) to illustrate the spread of individual events, while combined localizations were rendered as gold spheres (10 nm) to indicate the most likely positions of fluorophores.

Wide-field single-molecule localization microscopy

Optical setup description

Wide-field measurements were performed using a custom-built optical setup, described elsewhere[2] and shown in Figure S1. A pulsed super-continuum white light laser (WL Laser) (SuperK Fianium, NKT Photonics) was used for sample excitation. A variable filter (VF) (SuperK Varia, NKT Photonics) connected to the laser output enabled flexible selection of the output wavelength (605-652 nm for efficient excitation of JF635b and Alexa Fluor 647). A neutral density filter (NE10A-A, Thorlabs), in tandem with a variable neutral density filter (ND) (NDC-50C-4-A, Thorlabs), was used to adjust the laser excitation power.

The laser beam was coupled into a single-mode optical fiber (SMF) (P1-460B-FC-2, Thorlabs) with a typical coupling efficiency of 40%. After exiting the fiber, the collimated laser beam was expanded 3.6× using telescope lenses (TL1 and TL2). The typical excitation intensity at the sample was approximately 1 kW/cm², sufficient to switch the fluorophore (Alexa 647) from the bright to the dark state (see zoom-in box in Figure S1) in the presence of the blinking agent

cysteamine (30070, Sigma-Aldrich) at 50 mM in PBS. Identical imaging parameters were used for imaging of samples labeled with JF635b.

The laser beam was focused onto the back focal plane of the TIRF objective (UAPON 100X oil, 1.49 NA, Olympus) using an achromatic lens (L1) (AC508-180-AB, Thorlabs). Beam displacement relative to the optical axis for switching between EPI, HILO, and TIRF illumination schemes was achieved using a translation stage (TS) (LNR25/M, Thorlabs). Smooth lateral positioning of the sample was enabled by a high-performance two-axis linear stage (M-406, Newport). In addition, an independent one-dimensional translation stage (LNR25/M, Thorlabs) combined with a differential micrometer screw (DRV3, Thorlabs) was used to move the objective along the optical axis for focusing.

Spectral separation of the collected fluorescence light from the excitation path was achieved using a multi-band dichroic mirror (DM) (Di03 R405/488/532/635, Semrock), which directed the fluorescence light toward the tube lens (L2) (AC254-200-A-ML, Thorlabs). The field of view was physically limited in the emission path by an adjustable slit aperture (SP60, OWIS) positioned in the image plane. Lenses L3 (AC254-100-A, Thorlabs) and L4 (AC508-150-A-ML, Thorlabs) re-imaged the emitted fluorescence light from the slit onto an emCCD camera (iXon Ultra 897, Andor). Band-pass filters (BP) (BrightLine HC 692/40) were used to further block scattered excitation light. The total magnification of the optical system on the emCCD camera was 166.6×, resulting in an effective pixel size of 103.5 nm in the sample space. All experiments were conducted at 23 °C to ensure mechanical stability of the optical setup.

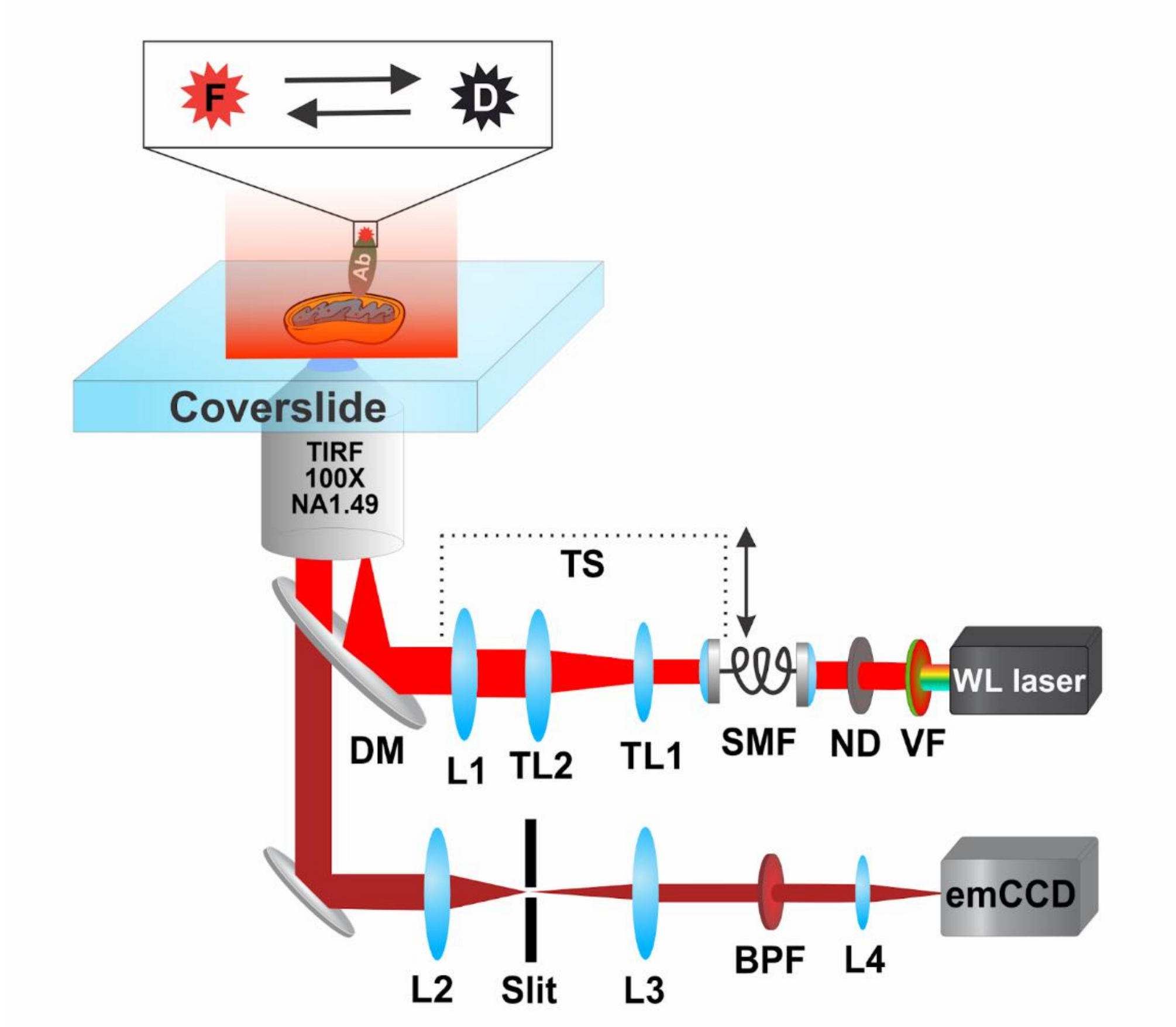

Figure S1. Schematics of a custom-built wide-field SMLM setup used for dSTORM imaging. The setup is equipped with a white-light laser source, enabling flexible excitation of fluorophores across different spectral regions, and a scientific emCCD camera with single-molecule sensitivity. It supports imaging using EPI, HILO, and TIRF excitation schemes.

Experimental conditions and image analysis

The following imaging parameters were used for both dSTORM and sb-dSTORM: A laser power of 26 mW (measured at the back of the objective) was used to excite both Alexa Fluor 647 (in the presence of GLOX–MEA, 50 mM) and JF 635b (in PBS buffer). Time series of 30,000–50,000 frames were recorded using a gain of 500 and an exposure time of 30 ms. The pixel size in the image plane was 103.5 nm.

The data were analyzed using the ImageJ plugin ThunderSTORM[44422222], and the super-resolved images were reconstructed at 10× magnification, yielding a pixel size of 10.35 nm. The reconstructed images were corrected for drift using the cross-correlation algorithm implemented in ThunderSTORM. Finally, filters were applied to reject out-of-focus emitters and poorly fitted Gaussians.

Target	Antibody/ Nanobody	Dye	Laser [nm]	Laser Power [mW]	Buffer	Total number of Frames	Total imaging time [min]
Mitochondria	NbALFA	AF647	640	35	GLOX	50,000	25
Mitochondria	NbGFP	AF647	640	35	GLOX	50,000	25
Mitochondria	NbALFA	JF635	640	26	PBS	50,000	25
Mitochondria	NbGFP	JF635	640	26	PBS	50,000	25
Mitochondria	anti-ALFA Ab	AF647	640	35	GLOX	50,000	25
Microtubule	αMouse Nb	JF635	640	26	PBS	50,000	25
Microtubule	αMouse Nb	JF635	640	26	Water	50,000	25
Vimentin	αRabbit Nb	JF635	640	26	PBS	50,000	25
PMP70	αRabbit Nb	JF635	640	26	PBS	50,000	25

Supplementary Table S1. Parameters used for Wide-field data acquisition.

Microtubules labeled with anti-mouse IgG1-JF635b – Images library

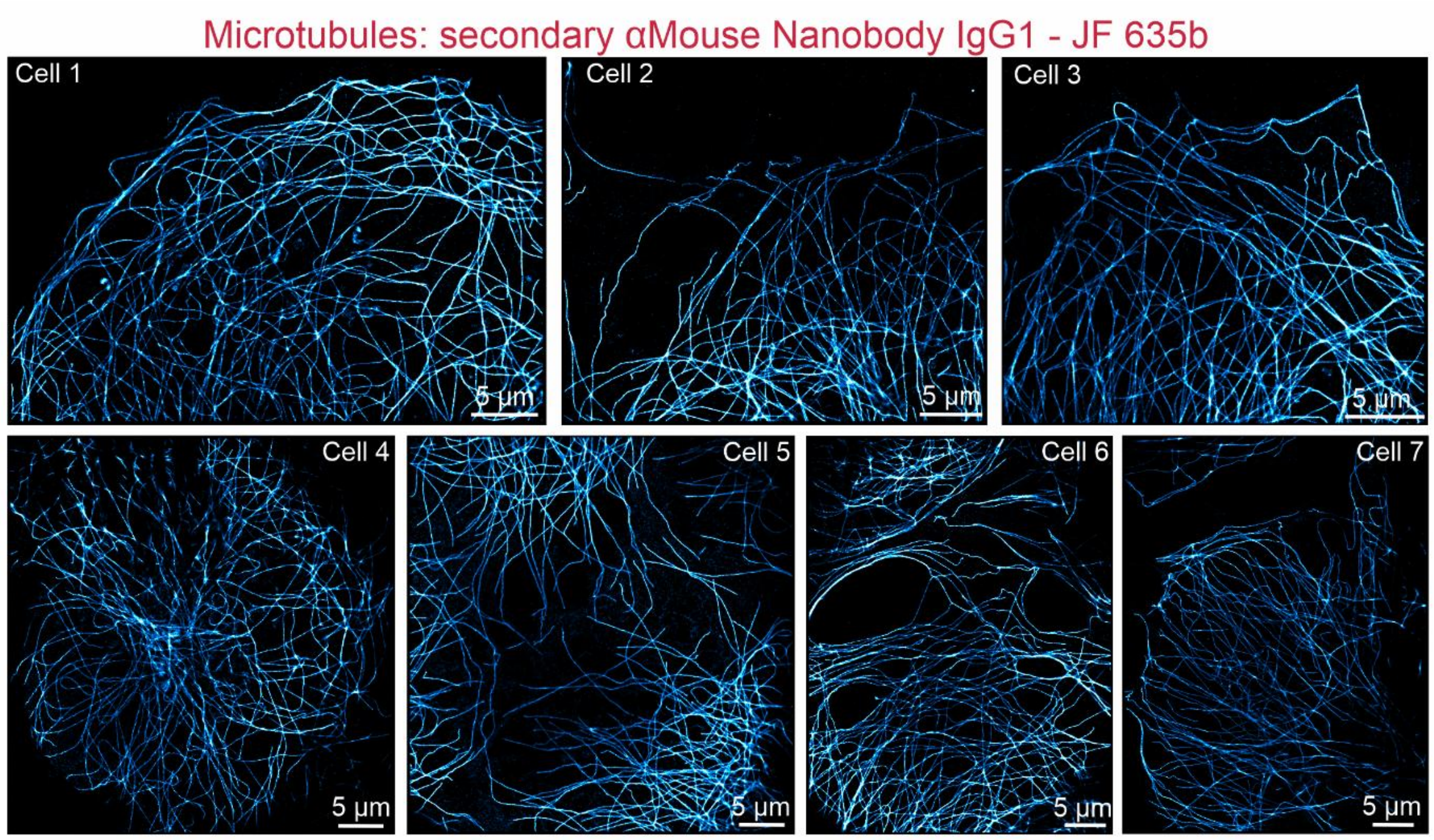

Figure S2. Library of sb-dSTORM images of microtubules labeled with JF635b in COS-7 cells imaged in PBS buffer. Representative wide-field sb-dSTORM reconstructions of microtubule networks acquired under identical imaging conditions. Images illustrate the consistency of labeling, filament continuity, and blinking performance across multiple cells and independent preparations.

Quantitative segmentation analysis of filamentous networks

The high localization density and continuity of the filament reconstructions obtained by sb-dSTORM enabled reliable quantitative segmentation analysis of both microtubule and vimentin networks. We emphasize that the purpose of this analysis is not to derive biological conclusions from the reported area distributions, which are expected to vary substantially across different cellular regions and cytoskeletal organizations. Rather, it serves as a representative example demonstrating that the image quality achieved with JF635b-labeled nanobodies is well suited for uncomplicated quantitative image analysis.

For microtubules, segmentation analysis yielded an area distribution with a mean value of 0.77 ± 8.89 μm^2, reflecting the heterogeneous organization of the filament network (Supplementary Fig. S4). Vimentin intermediate filaments exhibited a correspondingly narrow distribution with a smaller mean area of 0.15 ± 0.60 μm^2 (Supplementary Fig. S5), consistent with their denser network organization in COS-7 cells. The complete segmentation workflow is described in Fig. S3.

A custom Python-based pipeline was used to segment reconstructed super-resolution images. The workflow consisted of the following steps:

1. **Calibration** – Images were loaded and scaled to the correct dimensions (10.35 nm/pixel).
2. **Pre-filtering** – For microtubule datasets, an initial intensity threshold of 1% was applied.
3. **Inversion** – Images were inverted to emphasize voids rather than filaments.
4. **Morphological opening** – An opening operation was applied using a 3-pixel disk filter.
5. **Void segmentation** – Voids were detected and labeled.
6. **Artifact removal** – Partial voids adjacent to image borders were excluded to avoid bias.
7. **Hole filling** – holes, corresponding to filament fragments, were filled in detected void regions in order not to bias the measurement of mesh spacing.
8. **Quantitative analysis** – Morphometric parameters were extracted, including void area distributions and major/minor axis lengths.

The Python code used for segmentation is available from the corresponding author upon reasonable request.

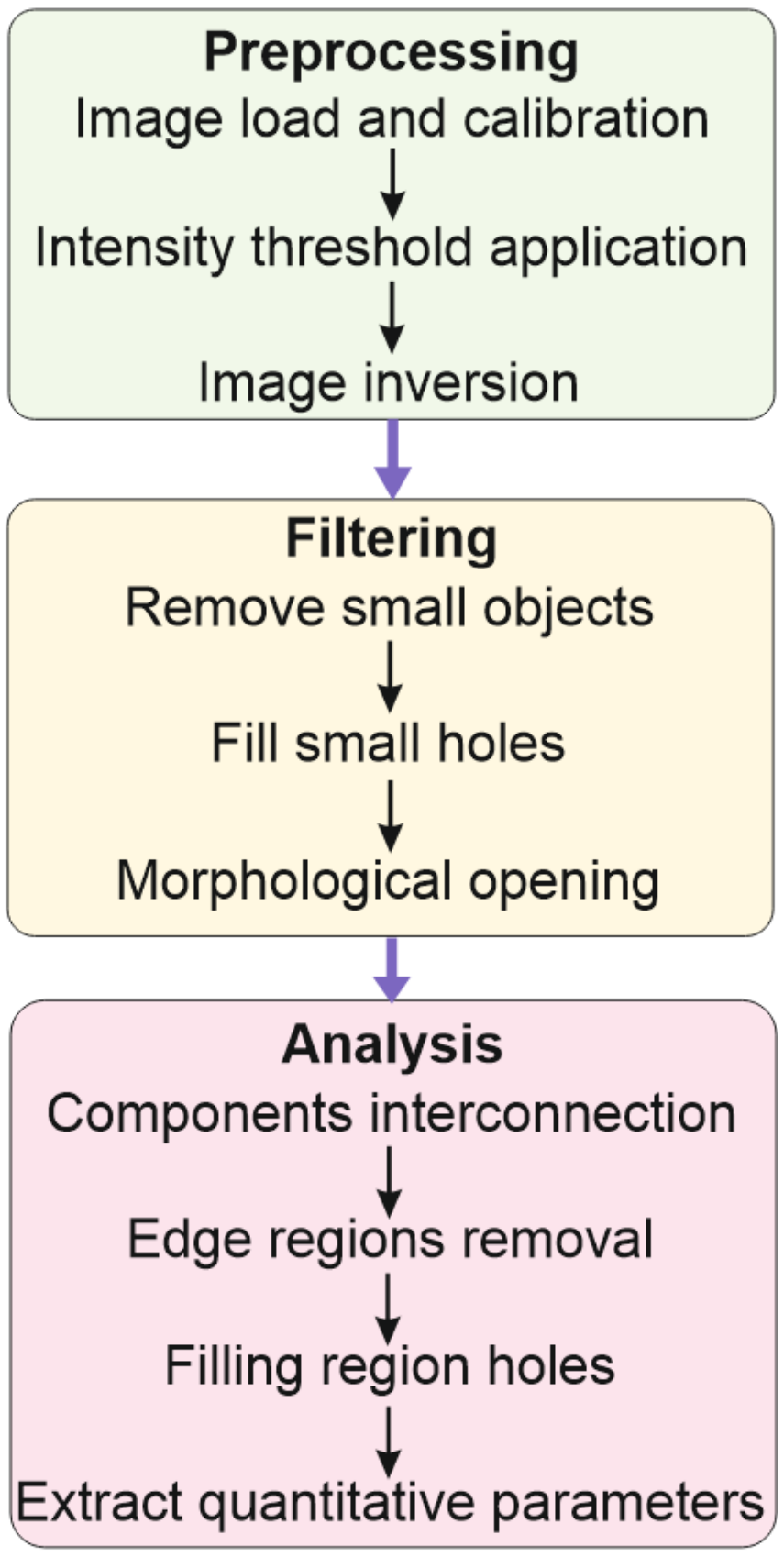

Figure S3. Schematics for the segmentation image analysis workflow.

Vimentin filaments labeled with anti-rabbit nanobody-JF635b – Images library

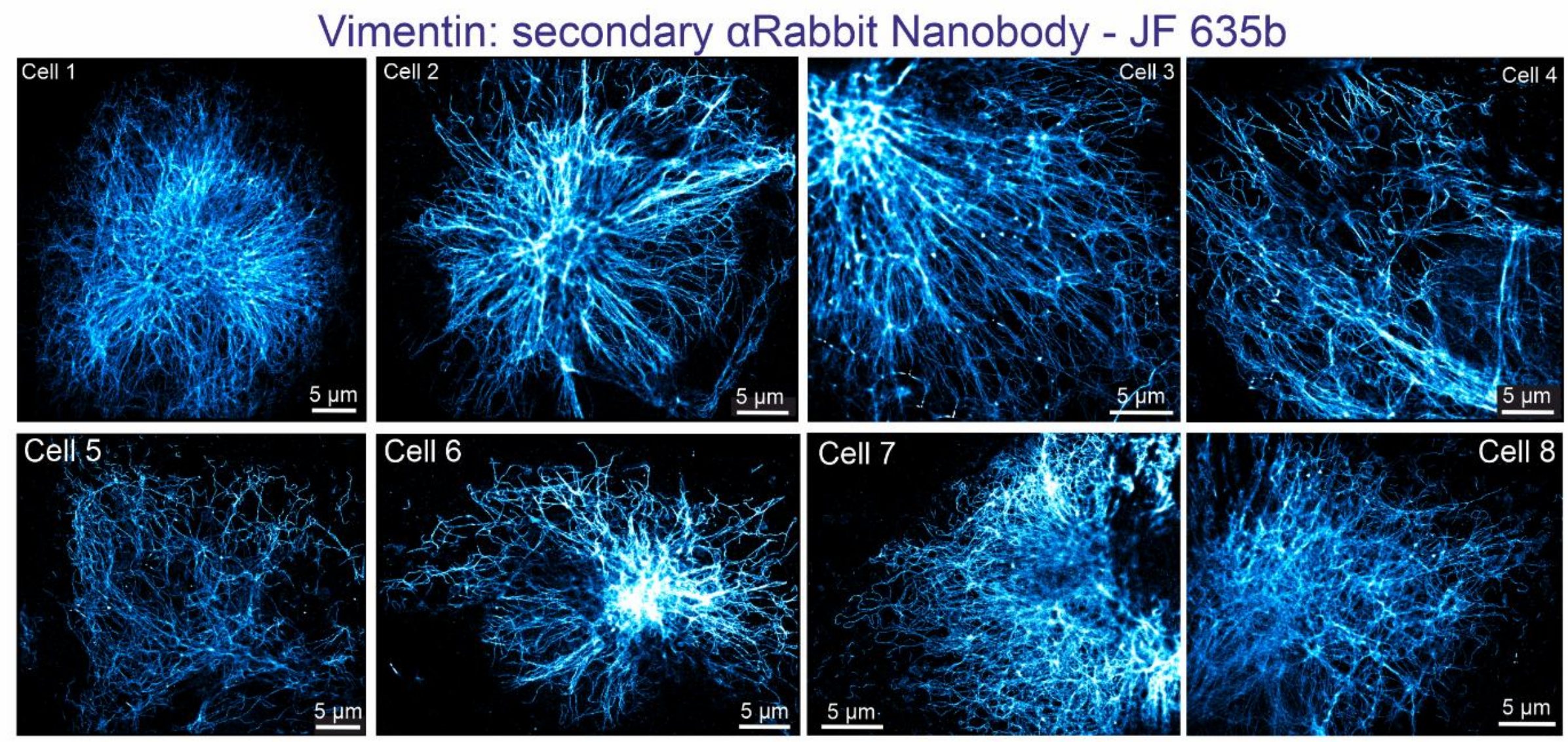

Figure S4. Library of sb-dSTORM images of vimentin labeled with JF635b in COS-7 cells imaged in PBS buffer. Representative wide-field sb-dSTORM reconstructions of vimentin intermediate filaments. The images highlight the dense filament networks, uniform labeling, and robust blinking behavior obtained with JF635b-conjugated nanobodies across samples.

Peroxisomes (PMP70) labeled with anti-rabbit nanobody-JF635b – Images library

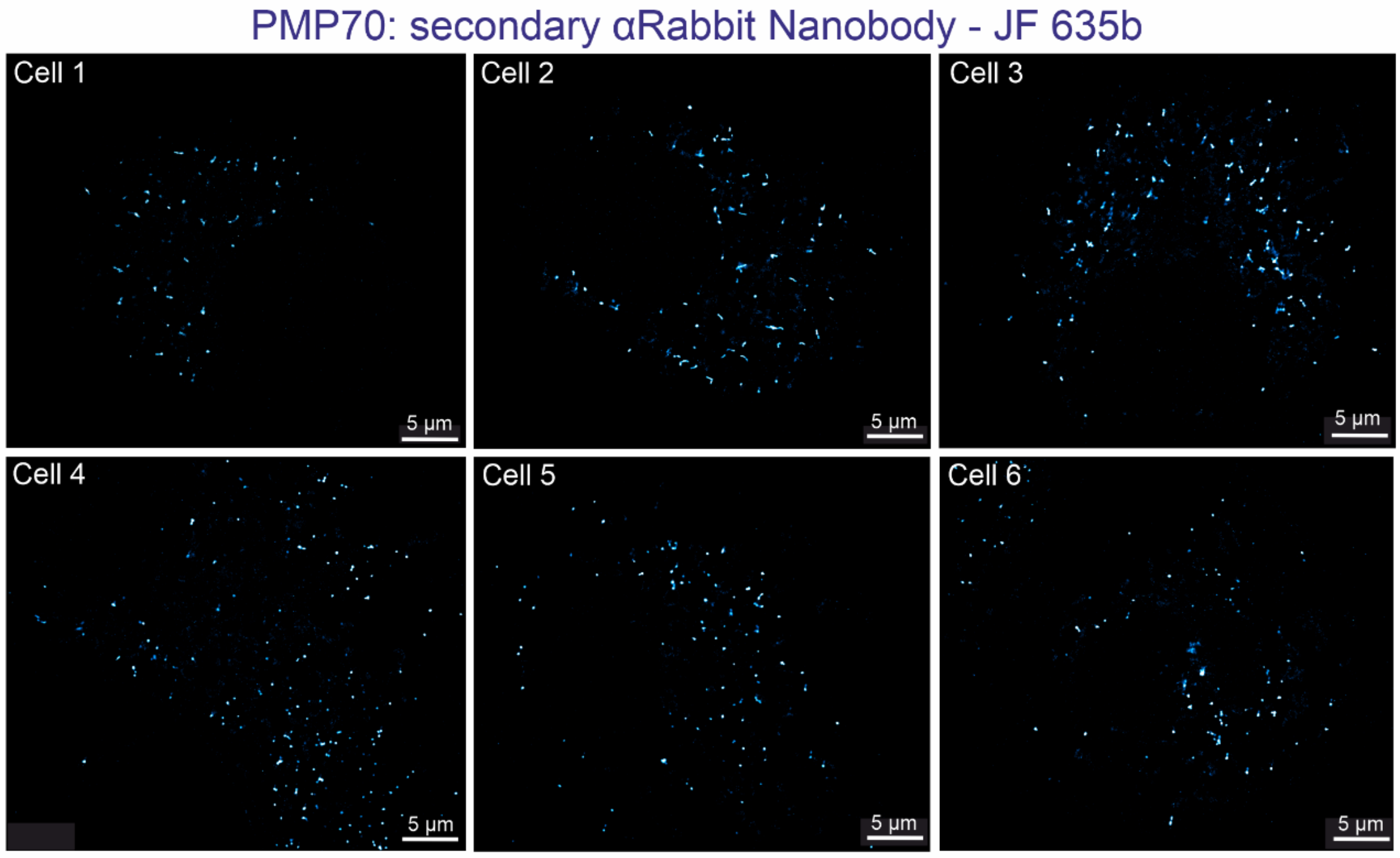

Figure S5. Library of sb-dSTORM images of PMP70-labeled peroxisomes in COS-7 cells imaged in PBS buffer. Representative sb-dSTORM reconstructions showing the morphology and distribution of PMP70-positive peroxisomes. The dataset demonstrates that JF635b performs reliably across different cellular targets, labeling strategies, and structural contexts.

Quantitative characterization of fluorophore performance

To complement the qualitative comparison presented in the main text, we performed a quantitative characterization of the photophysical performance of AF647- and JF635b-labeled probes across the representative datasets shown in the manuscript. For each dataset, the localization precision, average photon yield (normalized to 100 ms integration time), average blinking on-time, and characteristic photobleaching time constant were determined using the analysis procedures described in the Methods section.

The results are summarized in Supplementary Table S2. Together, these parameters provide a quantitative comparison of the blinking performance and photostability of the investigated fluorophores and support the conclusions presented in the main text.

Dataset	Labeling strategy	Loc. precision (nm)	Normalized photon yield (photons/ 100 ms)	On-time (ms)	Photobleaching time constant (s)
Fig. 1a upper	NbALFA–AF647	15.3	375	54	347.3
Fig. 1a bottom	NbGFP–AF647	14.1	327	58	334.1
Fig. 1b upper	NbALFA–JF635b	15.5	255	35	663.7
Fig. 1b bottom	NbGFP–JF635b	16.9	213	33	766.3
Fig. 1c	αMouse Ab-Ab–AF647	14.4	1159	50	739.7
Fig. 2a	αMouse Ab-Nb–JF635b	12.8	413	32	269.0
Fig. 2c	αRabbit Ab-Nb–JF635b	15.2	318	33	654.8
Fig. 3a (Fresh)	αMouse Ab-Nb–JF635b	16.0	736	34	614.5
Fig. 3a (3 months)	αMouse Ab-Nb–JF635b	17.0	203	35	503.0
Fig. 3a (6 months)	αMouse Ab-Nb–JF635b	17.7	392	39	456.7
Fig. 3a (12 months)	αMouse Ab-Nb–JF635b	17.9	258	37	334.1
Fig. 3b (water)	αMouse Ab-Nb–JF635b	15.9	573	38	938.9

Supplementary Table S2. Quantitative characterization of dSTORM images.

Antibodies and nanobodies used in this study

Supplementary Table S4 provides a complete overview of all primary and secondary antibodies and nanobodies used in this study, including their target, host species, fluorophore conjugation, supplier, catalog number, and the dilution factor used for labeling reaction.

Antibody or Nanobody	Vendor	Information	Dilution
FluoTag®-X2 anti-GFP (NbGFP; clone 1H1)	NanoTag Biotechnologies	Cat#: N0304	1:1000
FluoTag®-X2 anti-ALFA-tag (NbALFA)	NanoTag Biotechnologies	Cat#: N1502	1:1000
Mouse anti-alpha-Tubulin	Synaptic Systems	Cat# 302211	1:1000
Rabbit monoclonal anti-Vimentin	Abcam	Cat# ab92547	1:500
Recombinant anti-ALFA Antibody (mouse IgG1)	NanoTag Biotechnologies	Cat# N1582	1:500
Goat anti-Mouse IgG Cross-Adsorbed Antibody - AF647	ThermoFisher	Cat# A-21235	1:500
FluoTag®-X2 anti-Mouse IgG1	NanoTag Biotechnologies	Cat# N2002	1:500
FluoTag®-X2 anti-Rabbit IgG	NanoTag Biotechnologies	Cat# N2402	1:500

Supplementary Table S3. Antibodies and Nanobodies used for labelling.

Confocal fluorescence lifetime single-molecule localization microscopy

Optical setup description

CLSM measurements were performed using a custom-built confocal microscopy setup described previously[6]; see Figure S2. Briefly, a white light (WL) laser (Koheras SuperK Power) with a fixed repetition rate of 40 MHz was used. An acousto-optic tunable filter (AOTF) (Koheras SpectraK Dual) attached to the WL laser output provided full flexibility in selecting the excitation wavelength.

The laser beam was coupled into a single-mode fiber (SMF) (PMC-460Si-3.0-NA012 3APC-150-P, Schäfter+Kirchhoff) using a fiber coupler (60SMS-1-4-RGBV-11-47, Schäfter+Kirchhoff), and was decoupled and collimated using a 10× air objective (UPlanSApo 10×, 0.40 NA, Olympus). A quad-band dichroic mirror (DM) (ZT405/488/561/640rpc, Chroma) was used to direct the excitation light into the specimen and to separate it from the emission light. The excitation beam passed through a fast laser scanning system (FLIMbee, PicoQuant GmbH). The scanning system deflected the beam to image large regions of interest while maintaining the focus position by directing the laser beam onto the back focal plane of the objective (UApo N 100×, 1.49 NA oil, Olympus). The region of interest and focus plane were controlled by a manual XY stage (Olympus) and a z-piezo stage (Nano-ZL100,

MadCityLabs), respectively. The emitted fluorescence light was collected using the same objective and focused onto a pinhole with a 100 µm diameter (PH) (P100S, Thorlabs) using a 180 mm achromatic lens (L1) (AC508-180-AB, Thorlabs). The emission light was then collected and collimated by a 100 mm lens (L2) (AC508-200-A, Thorlabs). Long-pass filters (LP) (488 LP, 647 LP Edge Basic, Semrock) were used to block excitation laser light in the emission path. Band-pass filters (BP) (BrightLine HC 692/40, Semrock) was used to further reject scattered excitation light.

Finally, the emission light was focused onto a SPAD detector (SPCM-AQRH, Excelitas) using an achromatic lens (L3) (AC254-030-A-ML, Thorlabs). The output signal from the photon detector was recorded using a TCSPC system (HydraHarp 400, PicoQuant).

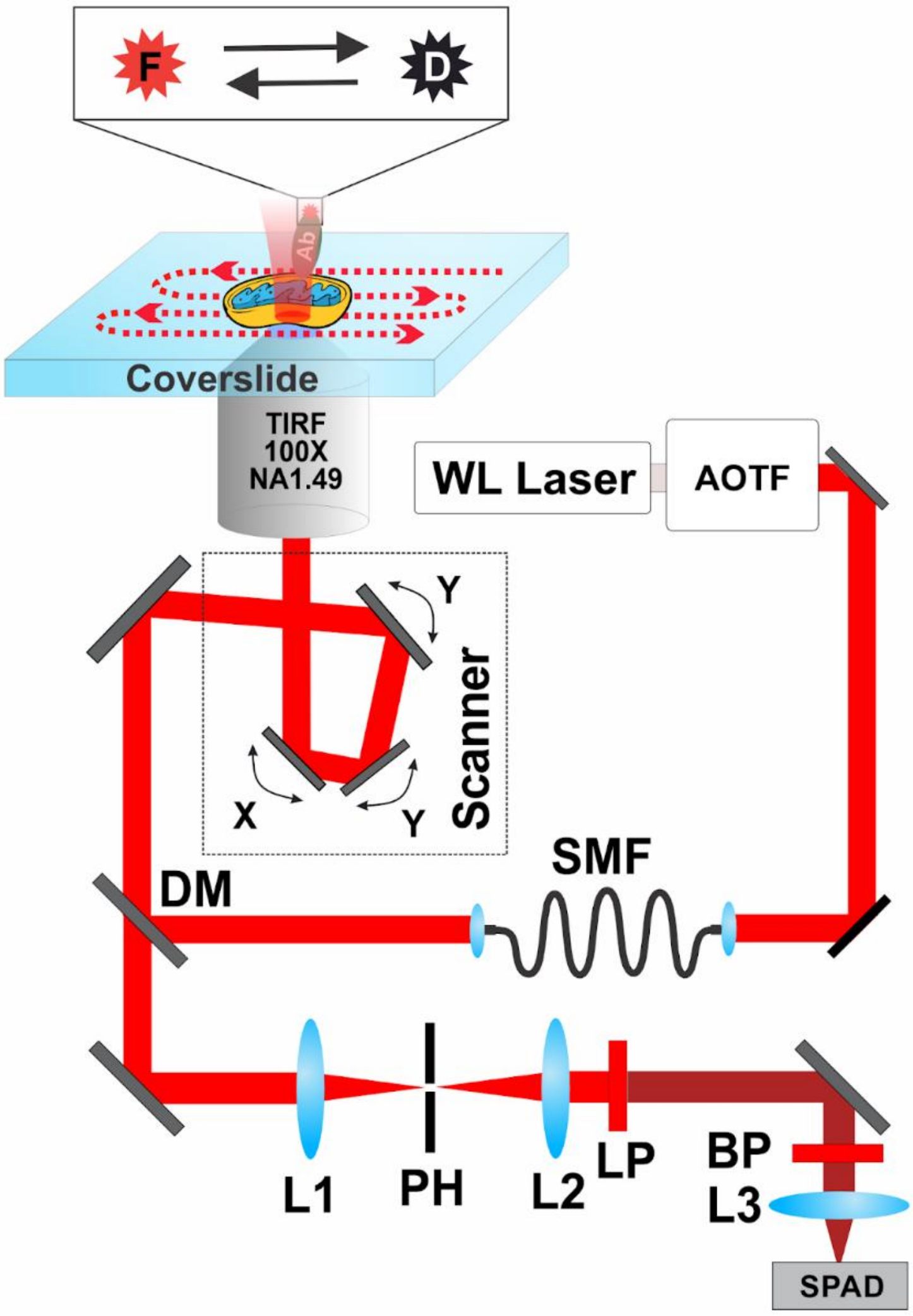

Figure S6. Schematic representation of the custom-built time-resolved CLSM optical setup. The setup is equipped with a white light laser source and a time-resolved modality.

Experimental conditions and image analysis

Data were acquired using commercial software (PicoQuant SymPhoTime 64), which controlled both the TCSPC and scanner systems. For CLSM-based FL-SMLM, regions of interest measuring 10 × 10 µm² were scanned. Each image consisted of four repeated scans of the region, with a virtual pixel size of 100 nm and a dwell time of 30 µs/pixel. A time series of 50.000 frames was recorded.

MINLFLUX imaging parameters

Parameter	MINFLUX iteration				
	Pre-local.	1	2	3	4
L (nm)	289	289	152	76	40
TCP	hexagon	hexagon	hexagon	hexagon	hexagon
# of photons	160	150	100	100	150
Laser power factor	1	1	2	4	6
TCP dwell time (ms)	≥1	≥1	≥1	≥1	≥1
Pattern repeat factor	1	5	5	5	5
CFR limit	2,00	0,50	2,00	0,80	2,00
Background threshold	15	10	10	10	10

Supplementary Table S4. Parameters used for MINFLUX data acquisition.

Bibliography

(1) Sograte-Idrissi, S.; Schlichthaerle, T.; Duque-Afonso, C. J.; Alevra, M.; Strauss, S.; Moser, T.; Jungmann, R.; Rizzoli, S. O.; Opazo, F. Circumvention of Common Labelling Artefacts Using Secondary Nanobodies. *Nanoscale* **2020**, *12* (18), 10226–10239. https://doi.org/10.1039/D0NR00227E.

(2) Sograte-Idrissi, S.; Oleksiievets, N.; Isbaner, S.; Eggert-Martinez, M.; Enderlein, J.; Tsukanov, R.; Opazo, F. Nanobody Detection of Standard Fluorescent Proteins Enables Multi-Target DNA-PAINT with High Resolution and Minimal Displacement Errors. *Cells* **2019**, *8* (1), 48. https://doi.org/10.3390/cells8010048.

(3) Schindelin, J.; Arganda-Carreras, I.; Frise, E.; Kaynig, V.; Longair, M.; Pietzsch, T.; Preibisch, S.; Rueden, C.; Saalfeld, S.; Schmid, B.; Tinevez, J.-Y.; White, D. J.; Hartenstein, V.; Eliceiri, K.; Tomancak, P.; Cardona, A. Fiji: An Open-Source Platform for Biological-Image Analysis. *Nat Methods* **2012**, *9* (7), 676–682. https://doi.org/10.1038/nmeth.2019.

(4) Ovesný, M.; Křížek, P.; Borkovec, J.; Švindrych, Z.; Hagen, G. M. ThunderSTORM: A Comprehensive ImageJ Plug-in for PALM and STORM Data Analysis and Super-Resolution Imaging. *Bioinformatics* **2014**, *30* (16), 2389–2390. https://doi.org/10.1093/bioinformatics/btu202.

(5) Basak, S.; Vu, K.-C.; Mougios, N.; Oleksiievets, N.; Pollack, Y. G.; Brandenburg, S.; Opazo, F.; Lehnart, S. E.; Enderlein, J.; Tsukanov, R. Versatile Microfluidics Platform for Enhanced Multitarget Super-Resolution Microscopy. *ACS Nano* **2026**. https://doi.org/10.1021/acsnano.5c18697.
(6) Thiele, J. C.; Helmerich, D. A.; Oleksiievets, N.; Tsukanov, R.; Butkevich, E.; Sauer, M.; Nevskyi, O.; Enderlein, J. Confocal Fluorescence-Lifetime Single-Molecule Localization Microscopy. *ACS Nano* **2020**, *14* (10), 14190–14200. https://doi.org/10.1021/acsnano.0c07322.
(7) Oleksiievets, N.; Mathew, C.; Thiele, J. C.; Gallea, J. I.; Nevskyi, O.; Gregor, I.; Weber, A.; Tsukanov, R.; Enderlein, J. Single-Molecule Fluorescence Lifetime Imaging Using Wide-Field and Confocal-Laser Scanning Microscopy: A Comparative Analysis. *Nano Lett.* **2022**, *22* (15), 6454–6461. https://doi.org/10.1021/acs.nanolett.2c01586.
(8) Sahl, S. J.; Matthias, J.; Inamdar, K.; Weber, M.; Khan, T. A.; Brüser, C.; Jakobs, S.; Becker, S.; Griesinger, C.; Broichhagen, J.; Hell, S. W. Direct Optical Measurement of Intramolecular Distances with Angstrom Precision. *Science* **2024**, *386* (6718), 180–187. https://doi.org/10.1126/science.adj7368.
(9) Ostersehlt, L. M.; Jans, D. C.; Wittek, A.; Keller-Findeisen, J.; Inamdar, K.; Sahl, S. J.; Hell, S. W.; Jakobs, S. DNA-PAINT MINFLUX Nanoscopy. *Nat Methods* **2022**, 1–4. https://doi.org/10.1038/s41592-022-01577-1.
